# PPO-GAT-Follow: Graph-Attention Reinforcement Learning for Robust Robot Person Following in Dense Crowds

**DOI:** 10.3390/s26154711

**Published:** 2026-07-24

**Authors:** Xinyu Zhou, Yongliang Shi, Songhao Piao, Chao Gao

**Affiliations:** 1 Multi-Agent Robot Research Center, Faculty of Computing, Harbin Institute of Technology, Harbin 150001, China; zhouxy@stu.hit.edu.cn; 2 Xinchen Qihang Inc., Beijing 100084, China; yongliang.shi@bdi.tech

**Keywords:** robot person following, crowd navigation, socially aware navigation, deep reinforcement learning, graph attention network, robot learning

## Abstract

**Highlights:**

**What are the main findings?**

- PPO-GAT-Follow improves RPF in dense crowds by explicitly modeling interactions among the follower robot, target pedestrian, and surrounding pedestrians.
- The task-oriented reward mechanism guides the policy toward a better task-level balance among target maintenance, visibility preservation, collision avoidance, rear position maintenance, proximity-aware social compliance, and action stability.

**What are the implications of the main findings?**

- Structured interaction representation and task-oriented reward design are effective for dense-crowd RPF under state-based geometric visibility loss with available target-relative pose estimates.
- The proposed framework shows zero-shot generalization across different crowd densities and can be integrated with localization, point cloud-based surrounding pedestrian perception and tracking, and velocity control modules under UWB-like target-relative pose input.

**Abstract:**

Robot person following (RPF) in dense crowds requires a mobile robot to maintain an appropriate relative position with respect to a moving target while avoiding surrounding pedestrians and satisfying rear-following and social constraints. This paper proposes PPO-GAT-Follow, an interaction-aware reinforcement learning framework for dense-crowd RPF under geometric visibility loss with available target-relative pose estimates. The follower, target pedestrian, and surrounding pedestrians are represented as graph nodes, and a graph attention encoder models their local interactions. A task-oriented reward mechanism jointly accounts for target maintenance, visibility preservation, collision avoidance, proximity-aware social compliance, rear position maintenance, post-arrival stabilization, and action stability. Experiments are conducted in IR-SIM under fixed-route and random-route settings, with comparisons against MPC, DWA, SFM, and an adapted SARL baseline. In the fixed-route setting with 12 background pedestrians, PPO-GAT-Follow achieves a task success rate of 98.8% and a collision rate of 1.1%, improving task success by 10.9 percentage points over MPC. In the random-route setting at the training density, it achieves 83.1% task success and an SPL of 0.815, outperforming MPC by 18.3 percentage points in task success; at this density, it also surpasses SARL in the main task-level metrics. Zero-shot evaluations across crowd densities, together with structural and reward ablations, reward weight sensitivity analysis, tolerance shift tests, multi-seed training, and stress testing under target pose noise and heterogeneous pedestrian dynamics, further demonstrate the effectiveness and reliability of the proposed framework. Gazebo-based validation also demonstrates system integration feasibility with localization, point cloud-based surrounding pedestrian perception, tracking, and UWB-like target-relative pose input. Nevertheless, visual target identification, re-identification, and perception-level occlusion recovery remain outside the scope of the present validation.

## 1. Introduction

Mobile robots are increasingly expected to operate in human-populated environments, such as campuses, hospitals, shopping malls, transportation hubs, care facilities, and public service areas. In these scenarios, navigation is no longer a purely geometric path-planning problem; instead, robots must move safely and efficiently while respecting human comfort, personal space, motion legibility, politeness, and other implicit social norms [1,2,3,4]. This line of research is commonly referred to as socially aware navigation, and its applications in pedestrian-dense environments are often studied as crowd navigation. For simplicity, this paper uses the term “crowd navigation” throughout.

Conventional crowd navigation mainly focuses on guiding a robot toward a static or predefined goal. However, many service scenarios require a robot to accompany a designated person, motivating robot person following (RPF). In RPF, a mobile robot must maintain an appropriate spatial relationship with a target pedestrian while safely interacting with surrounding pedestrians [5,6]. Typical applications include shopping mall service robots, hospital and care-facility assistive robots, logistics robots following workers, and guide robots in transportation hubs [6,7]. Unlike point-to-point navigation, RPF requires continuous relative position maintenance, target observability, and safe crowd interaction.

Although RPF already differs from standard point-to-point navigation, the problem becomes substantially more challenging when the robot operates in dense crowds. Compared with sparse environments, where the follower can often maintain a clear line of sight to the target and approach the desired following position with limited interference, dense pedestrian environments introduce frequent occlusions, restricted free space, complex human–human and human–robot interactions, and conflicts between target maintenance and collision avoidance [1,6,8]. The target may be partially or fully occluded by surrounding pedestrians, violating the full-observation assumption adopted by many RPF systems [9]. After the target reappears, visually similar pedestrians, viewpoint changes, and illumination variations may further cause re-identification ambiguity [10,11]. Therefore, RPF in dense crowds requires a careful balance between safety and target maintenance: overly aggressive following may reduce tracking error but increase collisions and close proximity exposure, whereas overly conservative behavior may improve short-term safety but lead to target loss or inefficient detours.

**Figure 1:**
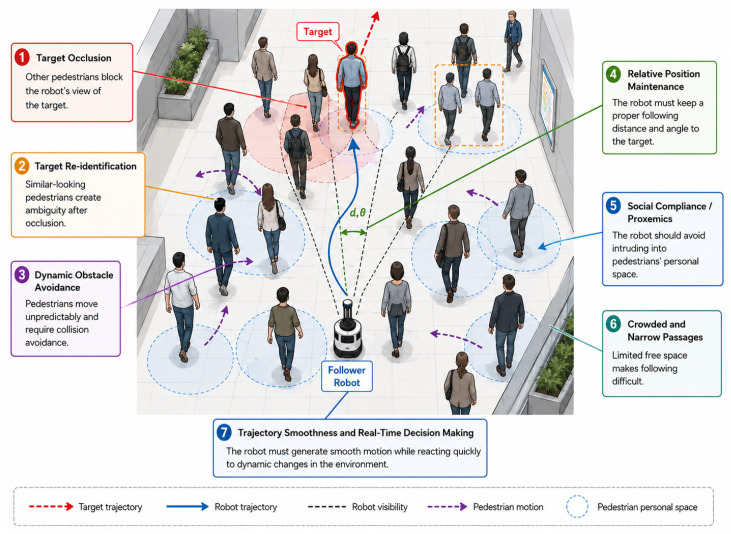
Key challenges of robot person following in dense pedestrian environments. The follower must track the target pedestrian while addressing occlusion, re-identification ambiguity, dynamic obstacle avoidance, relative position maintenance, social-compliance constraints, limited free space, and smooth real-time decision making. The red dashed arrow denotes the target trajectory, the blue arrow denotes the follower trajectory, black dashed lines indicate target visibility, purple arrows indicate pedestrian motion, and blue dashed ellipses represent pedestrian personal spaces.

As illustrated in Figure 1, RPF in dense crowds involves several coupled requirements. The robot must maintain an appropriate following distance and relative angle with respect to the target, rather than simply moving toward the target’s instantaneous position. Meanwhile, it must avoid dynamic pedestrians, respect proxemic constraints and personal spaces [3,4,12], handle temporary geometric visibility loss, and generate stable real-time actions. Abrupt or oscillatory motion may reduce motion predictability and social acceptability in human-populated environments [1,4]. These coupled requirements make RPF in dense crowds substantially more challenging than standard goal-reaching navigation.

Traditional local navigation methods, which generate short-horizon velocity commands or local trajectories based on the current robot state and nearby obstacles, have been widely used for robot navigation and collision avoidance. Representative examples include the Dynamic Window Approach (DWA) [13], the Social Force Model (SFM) [14], and Model Predictive Control (MPC)-based planners [15]. These methods are generally efficient and interpretable, and recent variants have incorporated social costs, pedestrian prediction, or risk-aware optimization for human-shared environments [16,17,18]. However, when applied to RPF in dense crowds, their performance is often limited by hand-crafted objective functions, simplified interaction assumptions, short-horizon decision making, or dependence on accurate pedestrian prediction. Specifically, sampling-based planners may overreact to immediate obstacles, force-based methods are sensitive to parameter tuning, and optimization-based planners depend heavily on prediction accuracy and cost-function design.

Deep reinforcement learning (DRL), which learns decision-making policies through trial-and-error interactions with simulated environments, provides a promising alternative to hand-crafted local planning methods [19,20,21]. Early DRL-based crowd-navigation methods mainly focused on decentralized collision avoidance or goal-reaching navigation in dynamic crowds. Subsequent studies improved interaction reasoning by introducing attention mechanisms, graph neural networks, recurrent models, and spatial–temporal representations, enabling robots to better capture human–robot and human–human interactions [22,23,24,25,26].

Other studies have incorporated dynamic feasibility constraints, velocity-obstacle-guided rewards, memory-based reasoning, and reward-shaping strategies to improve safety, efficiency, and social compliance [27,28,29,30,31,32]. These advances suggest the potential of DRL for RPF in dense crowds, where long-term interaction reasoning and multi-objective behavior balancing are both required.

Nevertheless, most existing DRL-based crowd-navigation methods are designed for goal-reaching tasks, where the robot moves toward a static destination while avoiding pedestrians. In contrast, RPF in dense crowds imposes additional task-specific constraints: the robot must continuously maintain a moving target relationship, jointly reason about the target and surrounding pedestrians, handle geometric visibility loss, and balance following accuracy with safety and proximity-aware social compliance. Although several studies have investigated robot following or guiding in crowded environments [6,7], DRL-based RPF under dense crowds with geometric visibility loss remains less explored. In particular, how to represent the interactions among the follower robot, target pedestrian, and surrounding pedestrians, and how to design rewards that jointly encourage target maintenance, visibility preservation, collision avoidance, proximity-aware social compliance, and stable motion remain open problems.

To address these challenges, this paper proposes PPO-GAT-Follow, an interaction-aware DRL framework for RPF in dense crowds. The proposed framework combines graph-based interaction modeling with task-oriented reward shaping to balance target maintenance, collision avoidance, proximity-aware social compliance, and stable following behavior. The main contributions are summarized as follows:

- We formulate dense-crowd RPF as an interaction-aware crowd-navigation problem that jointly considers target maintenance, local collision avoidance, and proximity-aware social compliance. A GAT-based representation is developed to model the relationships among the follower robot, target pedestrian, and surrounding pedestrians.
- We design a task-oriented reward mechanism that combines terminal rewards with step-wise dense shaping. The reward balances target maintenance, visibility preservation, collision avoidance, rear position maintenance, proximity-aware social compliance, post-arrival stabilization, and action stability.
- We conduct extensive IR-SIM experiments under fixed-route and random-route settings with different crowd densities. Comparisons with MPC, DWA, and an adapted SARL baseline, together with structural/reward ablations and reliability analyses, demonstrate the effectiveness and robustness of the proposed framework. SFM is retained as a force-based reference and analyzed separately due to its structural limitations under strict RPF completion conditions.
- We construct a Gazebo-based system integration validation pipeline combining localization, UWB-like target-relative pose input, point cloud-based surrounding pedestrian perception and tracking, and PPO-GAT-Follow control. This validation confirms the integration feasibility of the learned policy, while visual target identification, re-identification, and perception-level occlusion recovery remain outside the current scope.

The remainder of this paper is organized as follows. Section 2 reviews related work on DRL-based crowd navigation, traditional local planning methods, and RPF. Section 3 presents the proposed PPO-GAT-Follow framework, including the task formulation, graph-based state representation, GAT encoder, reward design, and PPO-based policy learning. Section 4 describes the experimental settings, including the simulation environment, episode randomization, visibility and occlusion handling, baseline methods, evaluation metrics, and ablation settings. Section 5 reports fixed-route and random-route performance, density generalization, following quality analysis, ablation studies, robustness and reliability analyses, qualitative trajectory comparison, and Gazebo-based system integration validation. Section 6 discusses the main findings, baseline interpretation, system integration implications, limitations, and future work. Section 7 concludes this paper.

## 2. Related Work

### 2.1. DRL-Based Crowd Navigation

Crowd navigation aims to enable mobile robots to move safely, efficiently, and acceptably in human-populated environments. Beyond geometric collision avoidance, it requires robots to consider human comfort, personal space, motion naturalness, and social acceptability [3,33]. Recent surveys show that this field has evolved from reactive obstacle avoidance and hand-crafted social rules toward human-centered motion generation, context-aware planning, and learning-based decision making [1,2,34].

Deep reinforcement learning (DRL) has been widely studied for crowd navigation because it can learn interaction-aware policies from simulated experience [35]. Early methods, such as CADRL and GA3C-CADRL, learned decentralized collision avoidance policies for multi-agent navigation [19,20]. Later studies improved crowd-level interaction reasoning through attention mechanisms, graph neural networks, recurrent models, and spatial–temporal representations. Representative examples include SARL for attention-based crowd reasoning [21], RGL for graph-based relation modeling [22], DS-RNN for spatial–temporal dependency modeling [23], and intention-aware methods for incorporating pedestrian intention prediction [26]. Other studies have explored velocity-obstacle-guided rewards, memory-based reasoning, and reward shaping to improve safety, efficiency, and social compliance [29,30,31,32].

However, most existing DRL-based crowd-navigation methods are still designed for goal-reaching tasks, where the robot moves toward a fixed destination while avoiding pedestrians. In contrast, RPF requires the robot to maintain a continuous spatial relationship with a moving target while avoiding surrounding pedestrians and respecting social constraints. Although recent work has begun to formulate socially aware RPF as a benchmark problem [6], RPF in dense crowds remains less explored than fixed-goal crowd navigation. Further investigation is therefore needed to balance target maintenance, occlusion handling, collision avoidance, rear position maintenance, and social compliance when robots follow a target pedestrian in dense crowds.

### 2.2. Traditional Local Planning Methods

Traditional local planning and obstacle-avoidance methods remain important baselines for mobile robot navigation because they are interpretable, computationally efficient, and easy to deploy. Representative categories include sampling-based reactive planners, force-based interaction models, and optimization-based planners. The Dynamic Window Approach (DWA) selects feasible velocity commands under robot kinematic and dynamic constraints using hand-crafted objectives such as heading alignment, obstacle clearance, and velocity preference [13,36]. Although socially aware variants have incorporated human-related costs for shared environments [16], DWA remains a short-horizon reactive method and tends to exhibit limited progress, local oscillations, or freezing-like behaviors in dense pedestrian interactions [37,38].

Force-based methods provide an intuitive formulation for navigation and collision avoidance. Artificial potential fields model goals and obstacles as attractive and repulsive forces [39], while the Social Force Model (SFM) describes pedestrian motion using human-inspired interaction forces [14]. These methods are lightweight and can encode interpersonal distance or social zone effects, but they are sensitive to parameter tuning and tend to suffer from local minima, oscillatory motion, or unrealistic interactions in dense crowds [40,41,42]. In RPF scenarios, force-based formulations may also overemphasize either attraction to the target or repulsion from surrounding pedestrians, making stable and safe following difficult.

Optimization-based planners, especially Model Predictive Control (MPC), formulate local navigation as a finite-horizon optimization problem and can explicitly incorporate robot dynamics, collision avoidance constraints, smoothness terms, and task-related costs [43]. Recent studies have combined MPC with control barrier functions, residual models, risk-aware optimization, or learning-based pedestrian prediction to improve safety and interaction awareness in crowded environments [15,17,18,44,45]. However, MPC-based methods often depend on accurate prediction, carefully designed cost functions, appropriate horizon selection, and tractable online optimization.

Overall, traditional local planning methods provide interpretable and deployable baselines, but they are mainly designed for fixed-goal navigation or local obstacle avoidance. RPF in dense crowds imposes additional requirements, including maintaining a desired relative position with a moving target, handling temporary occlusions, avoiding surrounding pedestrians, and respecting social spacing constraints. These coupled requirements are difficult to address using only hand-crafted rules or short-horizon optimization, motivating learning-based methods with interaction-aware representations.

### 2.3. Robot Person Following

Existing RPF studies have investigated different following configurations, including rear following, side-by-side following, frontal following, and adaptive following position selection [46,47,48]. Rear following is widely adopted because it is less intrusive and easier to maintain, whereas side-by-side following may provide more natural interaction but can introduce stronger conflicts with pedestrians and obstacles. To improve comfort and social acceptability, several studies have incorporated proxemic constraints and socially compliant behaviors into RPF systems [46,49,50].

From the motion-planning perspective, RPF methods have evolved from relative position tracking to local planning, optimization, and learning-based decision making. DWA-based methods generate feasible velocity commands under following and collision avoidance constraints [51], SFM-based methods model attractive and repulsive interactions among the robot, target, and surrounding pedestrians [52,53], and MPC-based methods optimize finite-horizon robot actions while considering target prediction, robot dynamics, and social space requirements [47,54]. Reinforcement learning-based methods have also been explored for human-following and follow-ahead tasks to address obstacle avoidance, occlusion avoidance, and frequent target direction changes [7,55,56].

Despite these advances, RPF in dense crowds remains insufficiently explored, as many existing studies are still evaluated in sparse or small-crowd scenarios [6]. In dense crowds, the robot must simultaneously maintain the target relationship, avoid surrounding pedestrians, handle target occlusion and re-identification, and respect social spacing constraints [9,11]. These coupled requirements motivate interaction-aware decision-making methods that balance target maintenance, safety, efficiency, and social compliance.

## 3. Materials and Methods

**Figure 2:**
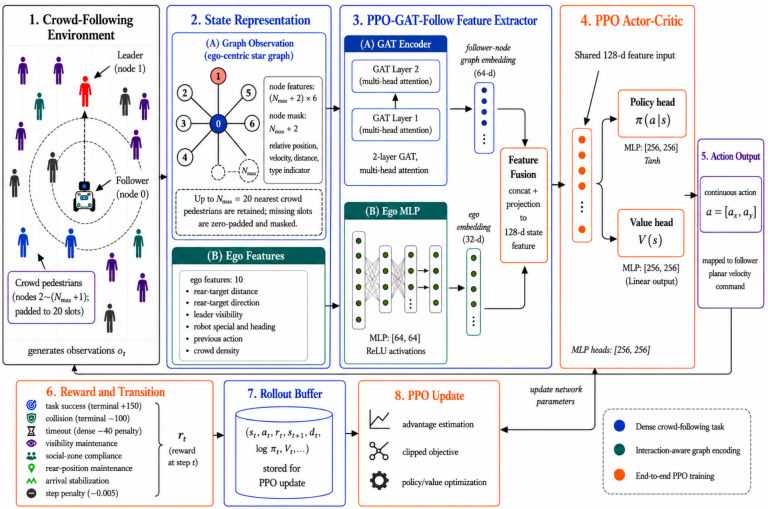
Overview of the proposed PPO-GAT-Follow framework. Graph-structured observations and ego-centric task features are encoded by separate branches and fused for PPO-based continuous control. The graph uses a follower-centered star topology with zero-padded background pedestrian slots and node masks to support variable crowd sizes.

We formulate RPF as an interaction-aware sequential decision-making problem and propose PPO-GAT-Follow, a PPO-based DRL framework that uses GAT to model interactions among the follower, leader, and surrounding pedestrians. As shown in Figure 2, graph-structured observations and ego-centric task features are encoded by the GAT and ego-feature branches, respectively, and then fused into a 128-dimensional latent representation. This representation is fed into the PPO actor–critic network to generate continuous planar velocity commands, while rollout transitions and rewards are used for policy updates.

The remainder of this section introduces the task formulation, graph-based state representation, GAT encoder, reward design, PPO learning process, and training configuration.

### 3.1. Crowd-Following Task and Environment

**Figure 3:**
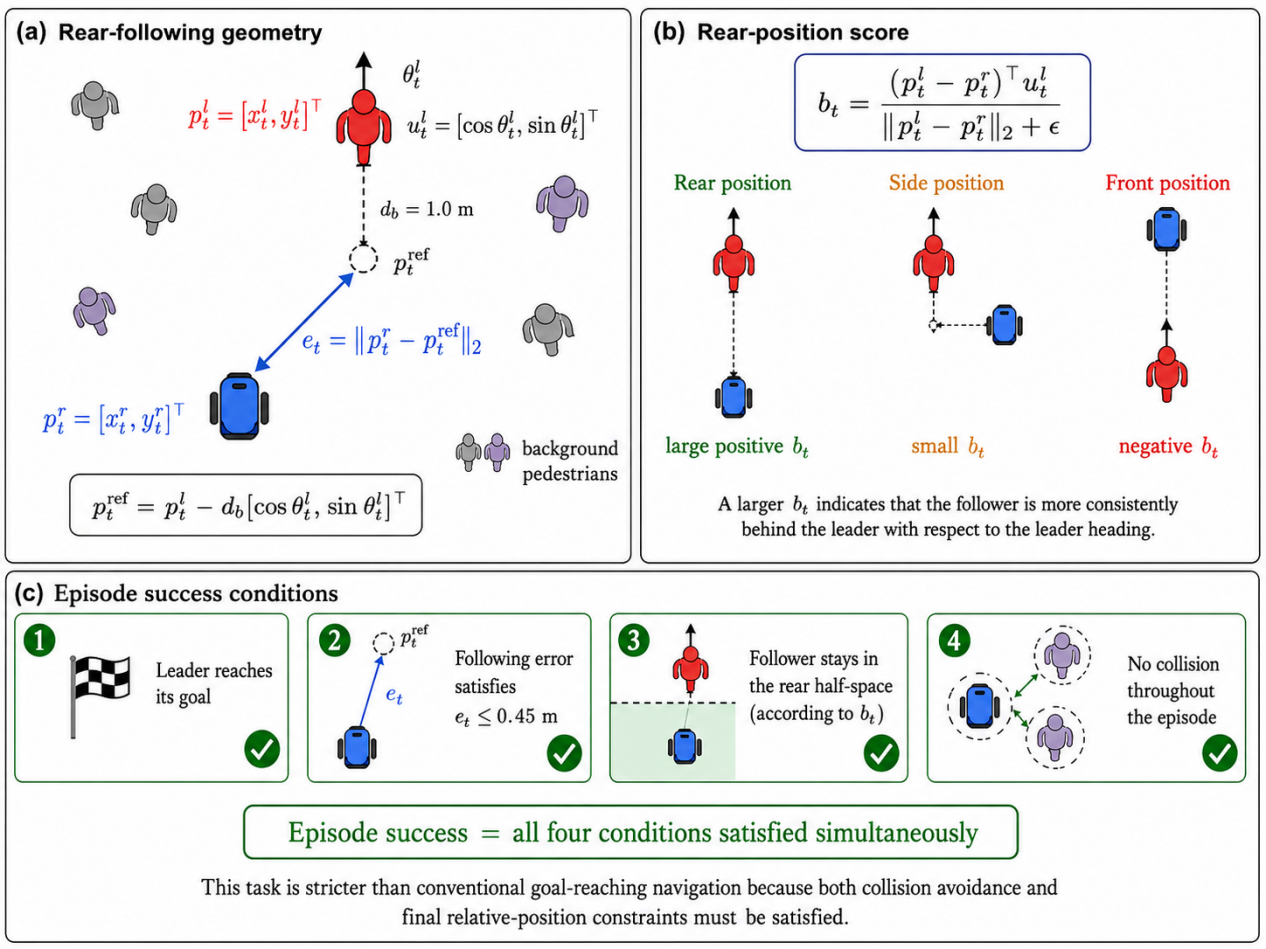
Visualization of the RPF task. (**a**) Rear-following geometry. (**b**) Rear position score ${\tilde{b}}_{t}$ for distinguishing rear following from side or frontal positioning. (**c**) Episode success conditions, including leader arrival, bounded following error, rear half-space satisfaction, low follower speed, and collision-free navigation.

The RPF task is defined in a two-dimensional crowd environment containing a follower robot, a designated target pedestrian, referred to as the leader in this paper, and multiple surrounding pedestrians, referred to as background pedestrians, as shown in Figure 3. The follower is required to accompany the leader until goal arrival while maintaining a rear-following position and avoiding collisions. Unlike conventional goal-reaching navigation, this task imposes both collision avoidance constraints with respect to background pedestrians and relative position constraints with respect to a moving leader.

Let $p_{t}^{r}={\left[x_{t}^{r},y_{t}^{r}\right]}^{⊤}$ and $p_{t}^{l}={\left[x_{t}^{l},y_{t}^{l}\right]}^{⊤}$ denote the positions of the follower robot and leader at time step *t*, respectively. The leader heading is denoted by $\theta_{t}^{l}$, and the desired rear-following distance is $d_{b}$. As shown in Figure 3a, the desired following reference is defined behind the leader along the opposite direction of its heading:

(1) $$p_{t}^{\mathrm{ref}}=p_{t}^{l}-d_{b}\left[\begin{array}{c} \cos\theta_{t}^{l}\\ \sin\theta_{t}^{l} \end{array}\right],\;$$

where $d_{b}=1.0\;m$ in all experiments. The following error is defined as

(2) $$e_{t}={∥p_{t}^{r}-p_{t}^{\mathrm{ref}}∥}_{2}.\;$$

This error evaluates whether the follower reaches the desired rear reference rather than merely staying close to the leader.

To distinguish rear following from simple distance keeping, the leader heading unit vector is defined as

(3) $$u_{t}^{l}=\left[\begin{array}{c} \cos\theta_{t}^{l}\\ \sin\theta_{t}^{l} \end{array}\right].$$

The rear half-space indicator is then given by

(4) $$\chi_{t}^{\mathrm{rear}}=I\;\left({\left(p_{t}^{l}-p_{t}^{r}\right)}^{⊤}u_{t}^{l}>0\right),\;$$

which is true when the follower lies behind the leader with respect to the leader heading. For reward shaping, we further define a continuous rear position score:

(5) $${\tilde{b}}_{t}=0.6\;\max\;\left(0,\;1-\frac{e_{t}}{2e_{\max}}\right)+0.4\;\mathrm{clip}\;\left(\frac{{\left(p_{t}^{l}-p_{t}^{r}\right)}^{⊤}u_{t}^{l}}{d_{b}+\epsilon },\;0,\;1\right),\;$$

where $e_{\max}=0.45\;m$ is the following position tolerance and ϵ is a small constant for numerical stability. The score ${\tilde{b}}_{t}\in \left[0,1\right]$ jointly measures rear reference accuracy and rear alignment. It is used only for reward shaping during training, rather than as a hard success condition.

As shown in Figure 3c, an episode is considered successful only if the leader reaches its goal, the follower satisfies $e_{t}\leq 0.45\;m$ and $\chi_{t}^{\mathrm{rear}}=1$, the follower speed is lower than $v_{\mathrm{stop}}=0.18\;m/s$, and no collision occurs throughout the episode. This definition is stricter than conventional point-to-point navigation because the robot must stop at a geometrically meaningful rear-following position after the leader arrives.

### 3.2. MDP Formulation and Learning Objective

The RPF problem is formulated as a Markov decision process (MDP), denoted by (S,A,P,R,γ). At each time step *t*, the follower observes a state $s_{t}\in S$, selects an action $a_{t}\in A$ according to the policy $\pi_{\theta }\left(a_{t}|s_{t}\right)$, receives a reward $r_{t}=R\left(s_{t},a_{t}\right)$, and transitions to the next state $s_{t+1}$ according to $P(s_{t+1}|s_{t},a_{t})$. The learning objective is to maximize the expected discounted return:

(6) $$J\left(\theta \right)=E_{\pi_{\theta }}\left[\sum_{t=0}^{T}\gamma^{t}r_{t}\right],$$

where γ is the discount factor and *T* is the maximum episode length.

The follower is modeled as a holonomic mobile robot. The policy outputs a two-dimensional normalized action:

(7) $$a_{t}={\left[a_{t}^{x},a_{t}^{y}\right]}^{⊤},\;a_{t}^{x},a_{t}^{y}\in \left[-1,1\right].\;$$

The action is then mapped to a planar velocity command in the world frame:

(8) $$v_{t}^{x}=a_{t}^{x}v_{\max},\;v_{t}^{y}=a_{t}^{y}v_{\max},\;$$

where $v_{\max}=1.2\;m/s$ denotes the maximum velocity component used for policy execution and benchmarking. In implementation, the follower is controlled by an omni-directional robot model. The world-frame velocity command is converted into a body-frame command, and the robot heading is updated to align with the motion direction. This implementation preserves holonomic motion capability while remaining compatible with the simulator interface.

### 3.3. Graph-Based State Representation

RPF in dense crowds requires the robot to jointly reason about the leader, surrounding background pedestrians, and possible target occlusions. Therefore, PPO-GAT-Follow adopts a graph-structured observation instead of a flattened state vector. At time step *t*, the graph is defined as

(9) $$G_{t}=\left(V_{t},E\right),$$

where $V_{t}$ is a fixed-size node set and E denotes a bidirectional star topology centered on the follower.

The graph contains $N_{\max}+2$ nodes. Node 0 represents the follower, node 1 represents the leader, and nodes $2,\ldots ,N_{\max}+1$ represent background pedestrians. If more than $N_{\max}$ pedestrians are observed, only the nearest $N_{\max}$ are retained; otherwise, unused slots are zero-padded and masked out. This fixed-size design enables the same network architecture to handle different crowd densities. In our implementation, $N_{\max}=20$, producing a 22×6 node feature matrix and a 22-dimensional node mask. For example, in the medium-density benchmark with 12 background pedestrians, only the corresponding pedestrian nodes are activated, while the remaining slots are padded and masked.

For each valid node *j*, the node feature is defined in the follower-centered coordinate frame:

(10) $$x_{t}^{j}=\left[\frac{\Delta p_{t,x}^{j}}{s_{c}},\frac{\Delta p_{t,y}^{j}}{s_{c}},\frac{v_{t,x}^{j}}{v_{\max}},\frac{v_{t,y}^{j}}{v_{\max}},\frac{∥\Delta p_{t}^{j}{∥}_{2}}{s_{c}},\tau_{j}\right],\;$$

where $\Delta p_{t}^{j}=p_{t}^{j}-p_{t}^{r}$, $s_{c}$ is the scene normalization scale, $v_{t}^{j}$ is the velocity of node *j*, and $\tau_{j}$ is the node-type indicator. The type indicator is set to 0.0 for the follower, 0.5 for the leader, and 1.0 for background pedestrians.

In addition to graph node features, an ego feature vector is used to encode follower-specific task information. Let $p_{t}^{\mathrm{tgt}}$ denote the active following target:

(11) $$p_{t}^{\mathrm{tgt}}=\left\{\begin{array}{cc} p_{t}^{\mathrm{ref}}, & \mathrm{if}\;\mathrm{the}\;\mathrm{leader}\;\mathrm{is}\;\mathrm{visible},\\ {\hat{p}}_{t}^{\mathrm{ref}}, & \mathrm{otherwise}, \end{array}\right.\;$$

where ${\hat{p}}_{t}^{\mathrm{ref}}$ is the last known rear-following reference maintained during geometric target occlusion. The ego feature is defined as

(12) $$g_{t}=\left[\frac{{∥p_{t}^{r}-p_{t}^{\mathrm{tgt}}∥}_{2}}{s_{c}},\cos\psi_{t},\sin\psi_{t},o_{t},\frac{∥v_{t}^{r}{∥}_{2}}{v_{\max}},\cos\theta_{t}^{r},\sin\theta_{t}^{r},a_{t-1}^{x},a_{t-1}^{y},\frac{n_{t}}{N_{\max}}\right],\;$$

where $\psi_{t}$ is the direction from the follower to $p_{t}^{\mathrm{tgt}}$, $o_{t}\in \left\{0,1\right\}$ indicates leader visibility, $\theta_{t}^{r}$ is the follower heading, $a_{t-1}$ is the previous action, and $n_{t}=\min(|P_{t}\left|,N_{\max}\right)$ is the number of valid background pedestrian nodes. Here, $P_{t}$ denotes the set of observed background pedestrians at time step *t*. When the leader is visible, the first ego feature corresponds to the normalized following error. When the leader is occluded, it represents the distance to the last known rear-following reference, providing a simple target memory cue. Invalid background pedestrian slots are zero-padded and suppressed by the node mask $M_{t}$ before GAT encoding.

In the main IR-SIM experiments, the leader-related state used in the observation is obtained from the simulator. When the leader is visible according to the geometric line-of-sight test, the current rear-following reference is used. When the leader is geometrically occluded, the last known rear-following reference is maintained as a simple memory cue. This design isolates the decision-making problem of following and collision avoidance, but it does not model visual detection uncertainty, re-identification failure, or tracking drift.

### 3.4. Graph Attention Encoder and Feature Fusion

The graph observation is encoded using a graph attention network (GAT). The edge set follows a follower-centered bidirectional star topology:

(13) $$E=\{\left(0,j\right),\left(j,0\right)|j=1,\ldots ,N_{\max}+1\},\;$$

which connects the follower with the leader and all valid background pedestrian nodes. This topology reflects the follower-centered decision process and enables the policy to assign different importance to neighboring agents.

For the *l*-th GAT layer, the attention coefficient from node *j* to node *i* is computed as

(14) $$\alpha_{ij}^{\left(l\right)}=\frac{\exp\left(\mathrm{LeakyReLU}\left(a_{l}^{⊤}\left[W_{l}h_{i}^{\left(l\right)}∥W_{l}h_{j}^{\left(l\right)}\right]\right)\right)}{\sum_{k\in N(i)}\exp\left(\mathrm{LeakyReLU}\left(a_{l}^{⊤}\left[W_{l}h_{i}^{\left(l\right)}∥W_{l}h_{k}^{\left(l\right)}\right]\right)\right)},\;$$

where $h_{i}^{\left(l\right)}$ is the hidden feature of node *i*, $W_{l}$ is a learnable linear projection, $a_{l}$ is the attention vector, ‖ denotes concatenation, and N(i) is the neighborhood of node *i*. The node feature is updated by

(15) $$h_{i}^{\left(l+1\right)}=\sigma \left(\sum_{j\in N(i)}\alpha_{ij}^{\left(l\right)}W_{l}h_{j}^{\left(l\right)}\right),\;$$

where σ(·) denotes a nonlinear activation function. Multi-head attention is used in the intermediate layer, and the final follower node embedding is taken as the graph-level interaction representation.

As shown in Figure 2, the GAT branch extracts follower-centered interaction features, while the ego MLP branch encodes task-specific ego features. The two embeddings are concatenated and projected into a latent representation:

(16) $$z_{t}=\phi_{\mathrm{proj}}\left(\left[\phi_{\mathrm{GAT}}{\left(X_{t},M_{t}\right)}_{0}∥\phi_{\mathrm{ego}}\left(g_{t}\right)\right]\right),\;$$

where $X_{t}$ is the node feature matrix, $M_{t}$ is the node mask, $\phi_{\mathrm{GAT}}{\left(\cdot \right)}_{0}$ denotes the follower node embedding, $\phi_{\mathrm{ego}}\left(\cdot \right)$ denotes the ego MLP, and $\phi_{\mathrm{proj}}\left(\cdot \right)$ projects the fused feature into a 128-dimensional latent representation. The network configuration is summarized in Table 1.

### 3.5. Reward Design

The reward is designed to balance task completion, target maintenance, visibility preservation, collision avoidance, proximity-aware social compliance, rear position maintenance, and post-arrival stabilization. Regular steps use dense shaping rewards, whereas task success, collision, and timeout are handled by terminal conditions. The overall reward is defined as

(17) $$r_{t}=\left\{\begin{array}{cc} +150, & \mathrm{if}\;\mathrm{the}\;\mathrm{task}\;\mathrm{is}\;\mathrm{successful},\\ -100, & \mathrm{if}\;a\;\mathrm{collision}\;\mathrm{occurs},\\ r_{t}^{\mathrm{dense}}-40, & \mathrm{if}\;\mathrm{the}\;\mathrm{episode}\;\mathrm{times}\;\mathrm{out},\\ r_{t}^{\mathrm{dense}}, & \mathrm{otherwise}. \end{array}\right.$$

For success and collision steps, only the corresponding terminal reward is returned. For timeout steps, dense shaping is first computed and then penalized by −40.

The dense reward is computed as

(18) $$\begin{array}{cc} r_{t}^{\mathrm{dense}}= & -5.0\frac{{∥p_{t}^{r}-p_{t}^{\mathrm{tgt}}∥}_{2}}{s_{c}}+4.0\frac{{∥p_{t-1}^{r}-p_{t-1}^{\mathrm{tgt}}∥}_{2}-{∥p_{t}^{r}-p_{t}^{\mathrm{tgt}}∥}_{2}}{s_{c}}+r_{t}^{\mathrm{vis}}+r_{t}^{\mathrm{zone}}+r_{t}^{\mathrm{prog}}\\ \; & +r_{t}^{\mathrm{behind}}+r_{t}^{\mathrm{arr}}-0.005. \end{array}\;$$

The first two terms penalize the distance to the active following target and reward progress toward it, while the constant term acts as a small step penalty.

The visibility reward encourages maintaining geometric leader visibility:

(19) $$r_{t}^{\mathrm{vis}}=\left\{\begin{array}{cc} +0.25, & \mathrm{if}\;\mathrm{the}\;\mathrm{leader}\;\mathrm{is}\;\mathrm{visible},\\ -0.10, & \mathrm{otherwise}. \end{array}\right.\;$$

The proximity penalty discourages excessively close interaction with the leader and background pedestrians. To avoid ambiguity caused by different agent radii, we use signed surface clearance rather than raw center-to-center distance. Let $\rho_{r}$, $\rho_{l}$, and $\rho_{i}$ denote the radii of the follower, leader, and background pedestrian *i*, respectively. The corresponding clearances are defined as

(20) $$c_{t}^{r,l}={∥p_{t}^{r}-p_{t}^{l}∥}_{2}-\left(\rho_{r}+\rho_{l}\right),$$

and

(21) $$c_{t}^{r,i}={∥p_{t}^{r}-p_{t}^{i}∥}_{2}-\left(\rho_{r}+\rho_{i}\right).$$

A negative clearance indicates geometric overlap, whereas a positive clearance represents the free space between the agent boundaries. The proximity penalty is then defined as

(22) $$r_{t}^{\mathrm{zone}}=-1.0\Delta t\;I\;\left(c_{t}^{r,l}<0.8\right)-4.0\Delta t\;I\;\left(\min_{i}c_{t}^{r,i}<0.55\right).\;$$

The two terms penalize close-range interaction with the leader and surrounding pedestrians, respectively.

The leader progress reward is applied before the leader reaches its goal:

(23) $$r_{t}^{\mathrm{prog}}=I\left(\neg \;\mathrm{leader}\;\mathrm{arrived}\right)\cdot 2.0\frac{d_{t-1}^{l,g}-d_{t}^{l,g}}{s_{c}},\;$$

where $d_{t}^{l,g}$ is the distance between the leader and its goal. The rear-following reward is based on the continuous rear position score ${\tilde{b}}_{t}$ defined in Equation (5):

(24) $$r_{t}^{\mathrm{behind}}=\left\{\begin{array}{cc} 0.8\;{\tilde{b}}_{t}, & \mathrm{if}\;\mathrm{the}\;\mathrm{leader}\;\mathrm{has}\;\mathrm{arrived},\\ 0.4\;{\tilde{b}}_{t}, & \mathrm{otherwise}. \end{array}\right.\;$$

After the leader arrives, an additional post-arrival reward encourages the follower to settle near the rear-following reference:

(25) $$r_{t}^{\mathrm{arr}}=I\left(\mathrm{leader}\;\mathrm{arrived}\right)\left[1.0\left(1-\frac{{∥p_{t}^{r}-p_{t}^{\mathrm{tgt}}∥}_{2}}{s_{c}}\right)-4.0\frac{∥v_{t}^{r}{∥}_{2}}{v_{\max}}-1.5{∥a_{t}∥}_{2}^{2}\right].$$

The velocity and action-magnitude penalties in Equation (25) are applied only after leader arrival to suppress oscillation near the final following position.

The reward weights were selected according to task priority, reward-scale normalization, and preliminary empirical tuning. First, terminal rewards are assigned larger magnitudes than dense shaping terms because task success, collision avoidance, and timeout prevention represent the primary episode-level outcomes. Specifically, successful completion is strongly rewarded, while collision and timeout are penalized to discourage unsafe or incomplete following behaviors. Second, dense shaping terms are scaled according to the scene scale and simulation time step when applicable, so that no single term dominates training only because of its numerical range. For example, distance-related terms are normalized by the scene scale $s_{c}$, progress terms are also scale-normalized, and proximity penalties are multiplied by the simulation time step Δt. Third, the relative weights of the dense terms were tuned through preliminary training runs to balance target maintenance, visibility preservation, proximity-aware social compliance, rear position maintenance, post-arrival stabilization, and action stability. These manually selected values are not claimed to be globally optimal; therefore, we further conduct reward weight sensitivity analysis in Section 5.5 to examine whether the learned policy remains stable under moderate reward weight perturbations.

Reward ablations are conducted by removing the visibility term, the proximity penalty term, or the rear-following term while keeping the evaluation metrics unchanged. This allows us to examine whether each reward component improves following performance rather than merely changing the evaluation criterion. The influence of this tolerance choice is further examined through tolerance shift evaluation without retraining in Section 5.5.

### 3.6. PPO-Based Policy Learning

The policy is trained using Proximal Policy Optimization (PPO), which stabilizes policy updates through a clipped surrogate objective. The probability ratio between the current and old policies is defined as

(26) $$\rho_{t}\left(\theta \right)=\frac{\pi_{\theta }\left(a_{t}|s_{t}\right)}{\pi_{\theta_{\mathrm{old}}}\left(a_{t}|s_{t}\right)}.\;$$

The clipped policy objective is then given by

(27) $$L^{\mathrm{CLIP}}\left(\theta \right)=E_{t}\left[\min\left(\rho_{t}\left(\theta \right){\hat{A}}_{t},\mathrm{clip}\left(\rho_{t}\left(\theta \right),1-\epsilon ,1+\epsilon \right){\hat{A}}_{t}\right)\right],\;$$

where ${\hat{A}}_{t}$ is the estimated advantage and ϵ is the clipping coefficient. The final loss combines the clipped policy loss, value loss, and entropy regularization:

(28) $$L\left(\theta \right)=-L^{\mathrm{CLIP}}\left(\theta \right)+c_{v}L^{V}\left(\theta \right)-c_{e}H\left(\pi_{\theta }\right),\;$$

where $c_{v}$ and $c_{e}$ denote the value loss and entropy coefficients, respectively.

### 3.7. Training Configuration

PPO-GAT-Follow is implemented using Stable-Baselines3 (version 2.8.0) [57] with a multi-input policy. The graph observation is encoded by the custom GAT feature extractor, and the fused latent representation is shared by the policy and value networks. The main training parameters are summarized in Table 2.

Additional independent training seeds are used in the training seed reliability analysis in Section 5.5.

## 4. Experimental Settings

### 4.1. Simulation Environment

The crowd-following benchmark is implemented in the IR-SIM [58] multi-agent simulator using a 20m×20m obstacle-free workspace and a 0.1s time step. Each episode is limited to 700 steps (70s) and terminates upon follower collision; incomplete episodes are counted as timeouts.

As summarized in Table 3, the leader and background pedestrians are controlled by IR-SIM’s RVO module, while the follower is controlled by the evaluated planner or policy. All methods are therefore tested under identical pedestrian dynamics.

Three crowd-density settings are constructed by varying the number of background pedestrians while keeping the follower–leader pair unchanged, as shown in Table 4. The medium-density setting with 12 background pedestrians is used as the default benchmark, while the low- and high-density settings are used for density generalization analysis and stress testing.

### 4.2. Episode Randomization

To ensure reproducibility and reduce overfitting to a specific crowd configuration, each episode uses deterministic randomization controlled by a base seed and the episode index:

(29) $${\mathrm{seed}}_{\mathrm{ep}}=\mathrm{base}\_\mathrm{seed}+1,000,003\times \mathrm{episode}\_\mathrm{idx}.\;$$

Two evaluation regimes are considered. In the *fixed-route* setting, the leader and follower start positions remain unchanged. In the *random-route* setting, the leader start and goal positions are resampled per episode, with path lengths of 6–16m and a 2m boundary margin. The follower is initialized approximately 1.8m behind the leader. In both settings, each background pedestrian receives an independent uniform offset in [−0.25,0.25]m along each axis.

The same randomization protocol is used for training and testing. During testing, each method is evaluated using 10 base seeds with 200 episodes per seed.

### 4.3. Visibility and Occlusion Handling

Leader visibility is determined by a geometric line-of-sight test between the follower and leader centers against background pedestrian disks enlarged by a 0.05m margin. If blocked, the leader is marked invisible. Classical controllers search around the last known reference, and an occlusion is considered successfully handled if the leader is re-acquired within 5s. Learning-based methods use no hand-crafted search rule and act from observations containing the last known leader state.

The IR-SIM occlusion model is state-based and geometric. The simulator provides the leader state, while occlusion affects only the visibility indicator and the use of the last-known rear-following reference. Thus, the benchmark evaluates decision making under geometric line-of-sight loss with available target state information, but not visual detection, re-identification, tracking drift, or perception-pipeline failure.

The main visibility and task completion parameters are summarized in Table 5.

### 4.4. Baseline Methods

PPO-GAT-Follow is compared with three representative classical navigation baselines and one adapted learning-based baseline:

- *MPC*: A model-predictive local planner that optimizes finite-horizon commands using tracking and collision avoidance costs.
- *DWA*: A sampling-based local planner that selects feasible velocities based on heading, speed, and obstacle clearance.
- *SFM*: A social force-based method combining target attraction and pedestrian repulsion. Since standard SFM does not explicitly enforce precise rear reference positioning, rear half-space satisfaction, or post-arrival stopping, it is used as a force-based reference rather than the primary baseline for performance claims.
- *SARL*: A representative attention-based DRL crowd-navigation baseline adapted to RPF by replacing its original static navigation goal with the dynamically updated rear-following reference behind the leader. The adapted SARL baseline retains its original joint-state observation, discrete action formulation, DQN-based value learning, ϵ-greedy exploration, and discomfort-aware value term. For fair comparison, the RPF task definition, crowd configuration, random leader protocol, training budget, discount factor, learning rate, evaluation seeds, and major reward scales are aligned with those of PPO-GAT-Follow, while SARL-specific algorithmic components are kept unchanged.

MPC is treated as the strongest classical task-oriented baseline because it explicitly incorporates tracking and collision avoidance costs and achieves non-trivial RPF success. Accordingly, the main comparisons focus on MPC and SARL, while DWA and SFM serve as representative sampling-based and force-based references.

### 4.5. Evaluation Metrics

The evaluation metrics assess task completion, safety, target visibility, search ability, efficiency, and social compliance.

- *Task Success Rate*: The percentage of episodes in which the leader reaches the goal, the follower reaches the required rear-following position, and no follower collision occurs. Since task success requires collision-free navigation in our setting, this metric jointly reflects task completion, rear position satisfaction, and collision-free following.
- *Collision Rate*: The percentage of episodes in which the follower collides with the leader or any background pedestrian.
- *SPL*: Success weighted by path length. For episode *i*, it is computed as
  (30) $${\mathrm{SPL}}_{i}=S_{i}\frac{L_{i}^{*}}{\max(L_{i},L_{i}^{*})},\;$$
  where $S_{i}\in \left\{0,1\right\}$ is the task success indicator, $L_{i}$ is the actual follower path length, and $L_{i}^{*}$ is the reference shortest path length.
- *Target Visibility Ratio*: The proportion of time steps in which the leader is visible to the follower.
- *Search Success Rate*: The proportion of occlusion events in which the leader is re-acquired within 5s.
- *Path Length*: The total trajectory length traveled by the follower during an episode.
- *Navigation Time*: The elapsed time of an episode before task success, collision, or timeout.
- *Close Proximity Time*: The accumulated time for which the follower–leader surface clearance is below 0.8m or the minimum follower–pedestrian clearance is below 0.55m:
  (31) $$T_{\mathrm{prox}}=\Delta t\sum_{t=1}^{T}I\left(c_{t}^{r,l}<0.8;\vee ;\min_{i}c_{t}^{r,i}<0.55\right).$$
  The logical OR avoids double counting when both conditions hold. This metric measures close-range exposure and should be interpreted jointly with task success and collision rate, since rear following inherently requires proximity to the leader.

Path length and navigation time are interpreted together with task success and collision rate, because failed or prematurely terminated episodes may also produce shorter paths or shorter durations. Close proximity time is used as the quantitative metric corresponding to the proximity-related social zone constraint.

### 4.6. Fixed-Route Experiment

**Figure 4:**
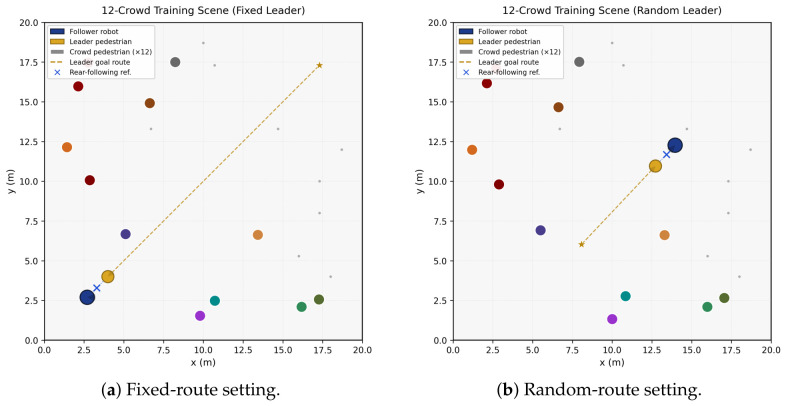
Top-down views of the training environments with 12 background pedestrians in IR-SIM. The follower, leader, and RVO background pedestrians share the same 20m×20m scene template. In the fixed-route setting, the leader follows a predefined start–goal route; in the random-route setting, the leader start and goal positions are resampled across episodes. The dashed line and star denote the leader route and goal, and the blue cross marks the rear-following reference point.

The fixed-route experiment evaluates performance along a repeatable leader trajectory. The leader and follower start positions remain fixed, while background pedestrians receive small initial perturbations. As shown in Figure 4a, the follower starts at (2.7,2.7,0), and the leader moves from (4.0,4.0,0.785) to (17.3,17.3,0.785). The random leader option is disabled during training and testing. PPO-GAT-Follow is trained for 2 million steps and evaluated over 10 seeds with 200 episodes each. The detailed training and testing settings for the fixed-route experiment are summarized in Table 6.

### 4.7. Random-Route Experiment

The random-route experiment evaluates generalization across varied leader trajectories. As shown in Figure 4b, the leader start and goal are resampled per episode, and the follower is initialized behind the leader. The random leader option is enabled during training and testing. This setting is more challenging because the policy must adapt to varying target directions, path lengths, and crowd interactions. The detailed training and testing settings for the random-route experiment are summarized in Table 7.

### 4.8. Ablation Settings

Ablations are conducted under the random-route setting using the full PPO-GAT-Follow model as reference. PPO-MLP-Follow replaces the GAT encoder with a flat observation, while GAT-1Layer reduces the graph encoder to one layer. Reward ablations remove one component at a time. All PPO-based variants are trained for 2 million steps with identical seeds, hyperparameters, scenarios, and evaluation protocols. The structural and reward ablation variants are summarized in Table 8.

## 5. Results and Analysis

PPO-GAT-Follow is evaluated through six experiment groups: (i) fixed-route performance; (ii) random-route performance and density generalization; (iii) structural and reward ablations; (iv) reward weight, tolerance shift, training seed, and stress robustness analyses; (v) qualitative trajectory comparison; and (vi) Gazebo-based system integration validation. Unless stated otherwise, results are averaged over 10 evaluation seeds with 200 episodes each. Baselines include MPC, DWA, SFM, and the adapted SARL method.

### 5.1. Fixed-Route Performance

Table 9 reports fixed-route performance with 12 background pedestrians. The leader follows an identical start–goal route across episodes, while pedestrian initial positions are perturbed. PPO-GAT-Follow uses a separately trained fixed-route checkpoint; thus, these results are analyzed independently of the random-route experiments in Section 5.2.

PPO-GAT-Follow achieves the best task-level performance, with 98.8% task success, a 1.1% collision rate, and an SPL of 0.987. Relative to MPC, it improves success by 10.9 percentage points and reduces collisions by 10.8 percentage points. DWA yields shorter paths and times but substantially lower success and higher collision rates.

Compared with SARL, PPO-GAT-Follow improves task success by 4.5 percentage points, reduces collisions by 4.3 percentage points, and increases SPL by 0.046, supporting the benefit of the proposed graph-based representation. SFM preserves visibility but fails the strict rear position and post-arrival conditions and is therefore treated only as a force-based reference.

PPO-GAT-Follow has higher close proximity time because successful rear following keeps the robot near the leader; this metric should therefore be interpreted jointly with task success and collision rate rather than as a direct measure of social violations. Overall, PPO-GAT-Follow provides the strongest fixed-route RPF performance among the evaluated methods.

### 5.2. Random-Route Performance and Density Generalization

In the random-route experiments, the leader start and goal are resampled per episode. PPO-GAT-Follow is trained with 12 background pedestrians and evaluated without retraining in scenes containing 6, 12, and 20 pedestrians. This protocol tests generalization to sparser and denser crowds under identical route configurations and episode seeds. The complete random-route results across the three crowd-density settings are reported in Table 10.

At the training density, PPO-GAT-Follow achieves 83.1% task success, a 16.7% collision rate, and the highest SPL of 0.815. It outperforms MPC, DWA, and SARL in the main task-level metrics, including a 5.5 percentage-point success gain and a 0.125 SPL improvement over SARL.

Without retraining, it reaches 92.0% success in the six-pedestrian setting. Under 20 pedestrians, SARL attains slightly higher success (66.5% vs. 64.8%), while PPO-GAT-Follow provides higher SPL, visibility, and search success. These results indicate effective, though incomplete, density generalization.

PPO-GAT-Follow also records higher close proximity time than SARL and DWA, reflecting a trade-off between rear-following accuracy and conservative spacing. SFM preserves visibility but fails the strict rear position and stopping conditions and is therefore treated only as a force-based reference.

### 5.3. Following Quality Analysis

Task success reflects complete RPF completion, including leader arrival, rear position accuracy, and collision avoidance, but does not fully describe following quality. We therefore also evaluate SPL, target visibility, and search success.

PPO-GAT-Follow achieves the highest SPL at all densities: 0.912, 0.815, and 0.628 for 6, 12, and 20 pedestrians, respectively. Despite degradation in denser crowds, it consistently outperforms MPC and DWA in SPL and collision rate, indicating a better balance among completion, efficiency, and safety.

Its visibility ratio decreases from 97.8% to 90.4%, while search success remains above 91% with 20 pedestrians. The larger decline in task success shows that dense-crowd RPF is also limited by access to the rear-following region and stable collision-free completion.

SFM maintains high visibility and search success but achieves zero task success because it does not explicitly enforce rear position, tolerance, or stopping constraints. It is therefore used only as a force-based reference.

### 5.4. Ablation Study

All ablation experiments are conducted under the random-route setting with 12 background pedestrians. Each variant is trained for 2 million steps using the same training configuration and evaluated over 10 evaluation seeds with 200 episodes per seed. The structural ablation compares the full two-layer GAT model (A0) with PPO-MLP-Follow without graph encoding (A1) and a single-layer GAT variant (A2). The reward ablation individually removes the visibility reward (R1), social zone penalty (R2), and rear-following reward (R3), while keeping the task definition and evaluation metrics unchanged.

As shown in Table 11, removing the GAT encoder causes the largest structural degradation. Compared with the full model, A1 reduces task success from 83.1% to 74.0%, increases collisions from 16.7% to 25.8%, and decreases SPL from 0.815 to 0.665, indicating that flattened observations inadequately represent follower–leader–pedestrian interactions.

The single-layer GAT performs better than A1 but remains inferior to the full two-layer model, with a 4.3 percentage-point reduction in task success and a 3.5 percentage-point increase in collisions. Its occasionally higher visibility or search success does not improve overall task completion.

Table 12 shows that all reward components contribute to performance. Removing the proximity penalty causes the largest degradation, reducing task success by 5.1 percentage points and increasing both collisions and close proximity time. Removing the rear-following reward weakens position maintenance and increases navigation time, while removing the visibility reward consistently reduces visibility, search success, task success, and SPL.

Overall, the full model provides the best task success, collision rate, SPL, and close proximity time, demonstrating the complementary benefits of graph-based interaction modeling and task-oriented reward design.

### 5.5. Robustness and Reliability Analysis

To assess policy reliability beyond the main benchmark, we conduct four analyses under the random-route setting. Reward-weight sensitivity evaluates dependence on the selected reward configuration. Tolerance-shift robustness tests whether the learned behavior generalizes to nearby success criteria without retraining. Training seed analysis examines reproducibility across independent initializations. Finally, stress testing introduces target pose noise, heterogeneous pedestrian speeds, perturbed RVO parameters, and mixed motion patterns, and evaluates the unchanged policy with 12 and 20 pedestrians.

Together, these experiments assess reward robustness, criterion generalization, training reproducibility, and performance under less idealized target state and crowd dynamics conditions.

#### 5.5.1. Reward-Weight Sensitivity Analysis

To evaluate reward weight sensitivity, we independently retrain several PPO-GAT-Follow variants in the 12-pedestrian random-route setting. The Base model uses the original configuration; Target-heavy, Safety-heavy, and Visibility-heavy increase the corresponding dense reward group by 20%; and Random-perturb scales all dense reward weights by factors sampled from Uniform(0.8,1.2). Each variant is trained for 2 million steps and evaluated over 10 seeds with 200 episodes each.

As shown in Table 13, Base achieves the best overall performance, with 83.1% task success and an SPL of 0.815. Visibility-heavy and Random-perturb remain close, reaching 81.0% and 80.2% success, respectively, with comparable visibility and search performance.

By contrast, Target-heavy and Safety-heavy reduce success to 73.2% and 65.0% and increase collisions and navigation time. Excessive emphasis on a single objective therefore disrupts the balance among tracking, safety, stabilization, and efficiency; stronger safety weighting does not necessarily improve episode-level safety.

Overall, the policy tolerates balanced perturbations but remains sensitive to large relative weight changes, supporting the original configuration as a more balanced design.

#### 5.5.2. Tolerance-Shift Robustness Analysis

To assess dependence on the training tolerance, the same PPO-GAT-Follow checkpoint is evaluated without retraining using final position tolerances of 0.35, 0.45, 0.50, and 0.60m, with all other settings unchanged.

As shown in Table 14, relaxing the tolerance to 0.50 or 0.60m causes only minor changes: task success increases from 83.1% to 83.6% and 84.0%, while SPL, visibility, search success, path length, and navigation time remain nearly unchanged. Thus, the policy does not simply exploit the original 0.45m threshold.

Under the stricter 0.35m criterion, task success decreases to 63.1%, while collisions and navigation time increase to 32.2% and 18.1s. Precise stabilization within a narrower rear-following region prolongs close interactions and increases conflict exposure. Overall, the policy is robust to moderate tolerance relaxation but more sensitive to stricter positioning requirements.

#### 5.5.3. Training Seed Reliability Analysis

To assess training seed reliability, five PPO-GAT-Follow policies are independently trained for 2 million steps under the same 12-pedestrian random-route setting and evaluated over 10 seeds with 200 episodes each.

As shown in Table 15, the policies achieve average task success of 79.7%±3.3%, collision rate of 19.0%±2.1%, and SPL of 0.777±0.041. Task success ranges from 75.5% to 83.1%, indicating moderate variation without performance collapse. Visibility and search success remain particularly stable at 0.951±0.002 and 0.952±0.009, respectively.

Although seed 42 yields the highest task success, the remaining policies retain comparable performance. The results indicate that PPO-GAT-Follow is not dependent on a single favorable initialization and exhibits reasonable reproducibility across independent training runs.

#### 5.5.4. Stress Robustness Test

To evaluate PPO-GAT-Follow under less idealized yet still controlled dense-crowd conditions, we conduct a stress robustness test without retraining. The same checkpoint trained in the default 12-pedestrian random-route setting is directly evaluated under the same random leader protocol. This experiment is intended to examine policy robustness when several benchmark assumptions are relaxed, rather than to rank all methods under a new benchmark.

The stress setting jointly introduces three types of perturbations. First, zero-mean Gaussian noise with σ=0.10m is added to the controller-facing leader pose and rear-following reference, while the clean simulator state is used only for evaluation. Second, pedestrians are assigned heterogeneous preferred speeds and per-episode RVO parameter jitter, with $v_{\max}∼U\left[0.6,1.1\right]\;m/s$, the avoidance factor sampled from U[1.5,3.0], and the neighbor range sampled from U[4,7]m, together with random crowd goals. Third, mixed motion models are used, including RVO-loop, SFM-loop, and wandering pedestrians, with ratios of 6/4/2 for 12 pedestrians and 10/7/3 for 20 pedestrians.

This experiment should therefore be interpreted as a stress test with noisier target input, heterogeneous crowd dynamics, and mixed motion patterns, rather than as a fully realistic human crowd benchmark. Evaluation is conducted at both 12- and 20-pedestrian densities using 10 seeds with 200 episodes per seed, and the original random-route results are included as references.

As shown in Table 16, performance decreases under the stress setting. With 12 pedestrians, task success drops from 83.1% to 75.0%, collision rate increases from 16.7% to 24.4%, and SPL decreases from 0.815 to 0.735. Visibility and search success decline only slightly, suggesting that the stress conditions mainly affect safe and precise completion rather than target observability.

With 20 pedestrians, task success decreases from 64.8% to 52.1%, collision rate rises from 33.7% to 46.5%, and SPL falls from 0.628 to 0.509. Shorter paths and times should not be interpreted as improved efficiency because unsuccessful or collision-terminated episodes may end earlier.

Despite these degradations, the unchanged policy retains 75.0% and 52.1% success in the 12- and 20-pedestrian settings, respectively. Thus, it remains functional under noisier target input and heterogeneous crowd motion, although these factors remain important limitations. Because other methods are not evaluated under the same stress conditions, this experiment assesses policy robustness rather than comparative superiority.

### 5.6. Qualitative Trajectory Analysis

**Figure 5:**
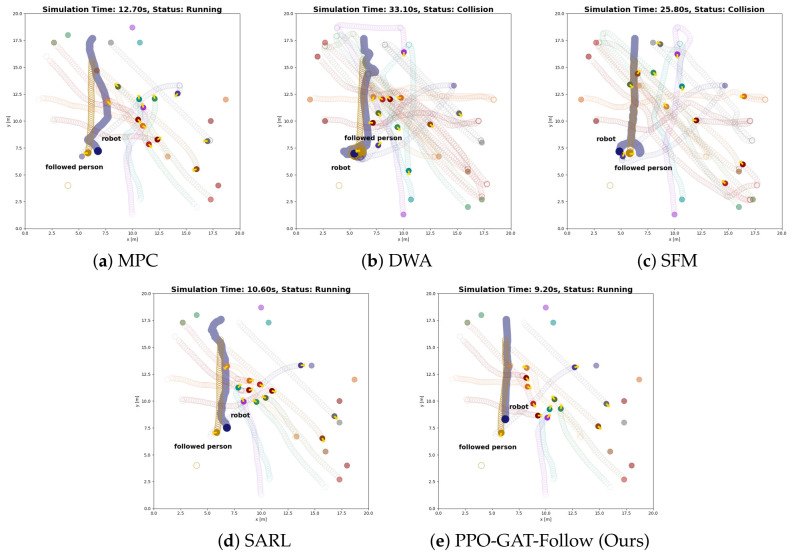
Representative trajectory comparison under the random-route setting with 12 background pedestrians. All methods are evaluated using the same random seed and episode index. The thick blue curve denotes the follower trajectory, the yellow curve denotes the leader trajectory, and the translucent curves denote the historical trajectories of the background pedestrians. The titles above the panels indicate the simulation time and episode status at the displayed instant.

To qualitatively illustrate the behavioral differences among the evaluated methods, Figure 5 presents representative trajectories from the random-route setting with 12 background pedestrians. All methods are evaluated using the same random seed and episode index, ensuring matched leader motion, pedestrian initialization, and crowd interaction conditions. The thick blue curve denotes the follower trajectory, the yellow curve denotes the leader trajectory, and the translucent curves show the historical trajectories of background pedestrians. The simulation time and current episode status are displayed above each panel. Additional dynamic demonstrations are provided in Supplementary Video S1.

The representative episode reveals distinct interaction patterns. MPC remains collision-free at the displayed instant but deviates broadly from the leader trajectory in the dense interaction region, reflecting conservative short-horizon optimization and delayed recovery to the desired rear-following position.

DWA repeatedly reacts to local crowd changes but fails to resolve the congestion and eventually collides. SFM also terminates in collision; although its attraction–repulsion mechanism maintains proximity to the leader, it does not explicitly enforce rear position accuracy or post-arrival stabilization.

Both learning-based methods show more coordinated behavior. SARL adapts to pedestrian flow but exhibits a larger trajectory deviation before returning toward the leader path. PPO-GAT-Follow follows a more compact route, avoids the densest pedestrian flow, and maintains the rear-following relationship with limited lateral deviation.

This episode is qualitative rather than statistical evidence, but it is consistent with Table 10: learning-based methods achieve more reliable completion than the classical baselines, while PPO-GAT-Follow provides a favorable balance among rear position maintenance, collision avoidance, and path efficiency.

### 5.7. Gazebo-Based System-Integration Validation

To evaluate system integration feasibility, PPO-GAT-Follow is deployed in a Gazebo Classic 11 simulation pipeline combining robot localization, UWB-like target-relative pose input, surrounding pedestrian perception and tracking, and policy-based control. This experiment assesses integration with robotic sensing and control modules rather than a complete visual RPF system.

The UWB-like interface directly provides the target pose relative to the robot and therefore excludes visual detection, re-identification, tracking drift, and perception-level occlusion recovery. The evaluation consequently focuses on policy execution, pedestrian perception, and collision avoidance with available target-relative pose estimates.

**Figure 6:**
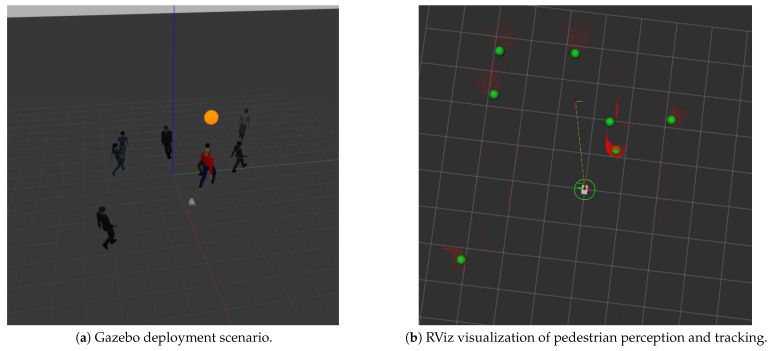
Gazebo-based system integration validation. (**a**) Simulation environment containing the robot, target pedestrian, and surrounding pedestrians. (**b**) RViz visualization of point cloud-based surrounding pedestrian detection and tracking. The red points denote the filtered Livox point cloud, the green circles denote detected pedestrian candidates.

As shown in Figure 6, data from a simulated Livox Mid-360 LiDAR (Livox Technology Co., Ltd., Shenzhen, China) are processed by Super-LIO [59] for localization. The robot, target, and pedestrian states are converted into the graph observation, from which PPO-GAT-Follow generates continuous velocity commands. Dynamic demonstrations are provided in Supplementary Video S1.

Surrounding pedestrians are estimated from Livox point clouds using ROI cropping, voxel downsampling, RANSAC ground removal, adaptive Euclidean clustering, and 3D size filtering. Detected candidates are tracked by a two-dimensional constant-velocity Kalman filter, and their positions and velocities are published through /pedestrian_state for policy inference. As shown in Figure 6b, this module converts raw point clouds into structured crowd state inputs.

The complete pipeline integrates Super-LIO localization, UWB-like target-relative pose input, pedestrian perception, graph-based inference, and velocity control. Gazebo Classic 11 and RViz2 (ROS 2 Humble) demonstrations confirm that PPO-GAT-Follow can execute closed-loop following and avoidance within an integrated three-dimensional simulation system.

These results demonstrate system integration feasibility under simulated sensing and available target-relative pose estimates. They do not validate a complete visual RPF system or robustness to real-world target occlusion, since visual identification, re-identification, tracking failure, and perception-level recovery are not evaluated.

## 6. Discussion

### 6.1. Influence of Crowd Density and Generalization

Crowd density strongly affects RPF performance. Increasing pedestrian numbers causes more frequent geometric occlusions, narrower collision-free passages, and stronger conflicts between rear position maintenance and avoidance, leading to degradation across all methods.

PPO-GAT-Follow is trained with 12 pedestrians and evaluated without retraining with 6 and 20 pedestrians. It performs better in sparse crowds but degrades under high density because the desired rear-following region is more often occupied or blocked. Nevertheless, it retains non-trivial success and competitive efficiency and observability.

Compared with SARL, PPO-GAT-Follow performs better at the training density. With 20 pedestrians, SARL achieves slightly higher success and marginally fewer collisions, whereas PPO-GAT-Follow maintains higher SPL, visibility, and search success. The two learning-based methods therefore show comparable robustness but different trade-offs.

The fixed-size masked graph supports moderate crowd-size variation without architectural changes, while follower-centered attention adapts to changing agent relevance. However, the high-density performance drop confirms that severe congestion remains challenging.

### 6.2. Interaction Modeling and Reward Shaping

Structural ablations confirm the importance of interaction-aware representation. Replacing the graph encoder with a flattened observation reduces task success and SPL while increasing collisions, navigation time, and close proximity time, indicating weaker modeling of follower–leader–pedestrian interactions.

The single-layer GAT outperforms the MLP variant but remains inferior to the two-layer model, suggesting that deeper attention aggregation improves the follower-centered representation. However, the star topology mainly captures direct follower–agent relations and does not explicitly model all pedestrian–pedestrian interactions.

Reward ablations show that RPF cannot rely on target attraction or visibility alone. Removing the visibility reward weakens observability, removing the rear-following reward degrades position maintenance and increases navigation time, and removing the proximity penalty causes the largest degradation in collisions and close proximity time.

Sensitivity analysis further shows that balanced perturbations are tolerated, whereas excessive emphasis on target- or safety-related rewards substantially degrades performance. Thus, the original coefficients provide a practical multi-objective balance rather than a globally optimal configuration.

### 6.3. Robustness, Reliability, and Remaining Trade-Offs

The robustness analyses examine sensitivity to reward weights, final position tolerance, training initialization, and less idealized operating conditions. Relaxing the tolerance from 0.45m to 0.50m or 0.60m causes only minor metric changes, indicating that the policy does not exploit the exact original threshold. By contrast, the stricter 0.35m criterion substantially reduces success and increases collisions and navigation time. Because this tolerance determines online success termination, stricter episodes continue longer and require stabilization within a narrower rear-following region, increasing exposure to later conflicts.

Independent training runs exhibit moderate variation without severe performance collapse, while visibility and search metrics remain stable, supporting reasonable reproducibility. Under target pose noise and heterogeneous crowd dynamics, performance declines, particularly with 20 pedestrians, but remains non-trivial. These results demonstrate policy-level robustness rather than robustness to realistic perception failures or human behavior.

Close proximity time also reveals a remaining trade-off. Because it combines task-required leader proximity with undesired pedestrian proximity, a high value does not directly imply unsafe behavior. PPO-GAT-Follow does not minimize this metric in every setting, as stronger rear position maintenance may increase close-range exposure. Future work will separate leader and pedestrian proximity components to distinguish accurate following from personal space intrusion.

### 6.4. Interpretation of Baseline Comparisons

The baselines should be interpreted according to their original objectives. MPC is the strongest classical task-oriented reference because it explicitly optimizes tracking and collision avoidance. DWA represents reactive short-horizon planning, while SARL provides an attention-based learning baseline.

The SARL comparison is particularly informative because both methods use aligned RPF task definitions, training budgets, reward scales, and evaluation protocols, while retaining their respective algorithm-specific action formulations. PPO-GAT-Follow performs better in the fixed-route and training-density settings, supporting the value of its graph representation and task-specific features. However, their comparable high-density results show that PPO-GAT-Follow does not dominate under every condition. Its advantage is therefore best characterized as a favorable overall balance among completion, safety, efficiency, and observability.

SFM requires separate interpretation because it does not explicitly enforce the rear reference tolerance, rear half-space constraint, or post-arrival stopping condition. Its zero task success reflects this structural mismatch rather than an inability to maintain target proximity or visibility. Accordingly, SFM is retained as a force-based reference, while the main quantitative comparisons focus on MPC and SARL.

### 6.5. Implications for Dense-Crowd RPF

The experiments show that target visibility and geometric reacquisition are necessary but insufficient for successful RPF. A method may preserve line of sight while still failing to reach the required rear-following position, avoid collisions, or stabilize after leader arrival.

Unlike conventional target tracking, RPF requires both target observability and appropriate relative positioning under dynamic crowd constraints. In dense scenes, the rear reference may be temporarily blocked, and direct pursuit can conflict with collision avoidance and social space preservation.

The proposed graph representation and task-oriented reward jointly address these requirements. Accordingly, dense-crowd RPF should be evaluated using complementary metrics, including task success, collision rate, SPL, target visibility, reacquisition success, navigation efficiency, and close proximity time, since no single metric fully characterizes following quality.

### 6.6. System-Integration Implications

The Gazebo validation demonstrates system integration beyond isolated 2D policy evaluation. The closed-loop pipeline combines simulated Livox Mid-360 LiDAR sensing, Super-LIO localization, UWB-like target-relative pose input, point cloud-based surrounding pedestrian perception, Kalman tracking, graph-based inference, and velocity control, showing that PPO-GAT-Follow can operate within a three-dimensional robotic simulation framework.

However, this experiment assumes directly available target-relative pose estimates and is not a complete visual RPF deployment. The designated target is not detected or identified through the point cloud perception pipeline, excluding visual detection, identity association, re-identification, and perception-level occlusion recovery.

Thus, the results support integration feasibility for perception, tracking, policy execution, and control, but do not validate robustness to target loss, identity switches, tracking drift, communication failure, or delayed state estimates.

### 6.7. Limitations and Future Work

Several limitations remain. First, the quantitative experiments rely mainly on IR-SIM with predefined RVO-based pedestrian dynamics. Although reproducible, this setting cannot fully capture abrupt motion changes, group behavior, cooperation, yielding, or socially dependent human responses. The stress tests add target pose noise and more diverse pedestrian dynamics, but remain controlled simulations rather than substitutes for real-crowd validation.

Second, the IR-SIM experiments use simulator-provided leader states, while the Gazebo system assumes a UWB-like target-relative pose interface. Thus, visual detection uncertainty, identity switches, re-identification failure, tracking drift, latency, and intermittent target loss are not evaluated. The Gaussian pose perturbations provide only preliminary robustness evidence and do not represent temporally correlated, biased, delayed, or discontinuous perception errors.

Third, PPO-GAT-Follow does not always minimize close proximity time. Stronger rear position maintenance and task completion may increase close-range interactions, revealing a remaining trade-off between strict following accuracy and social space preservation.

Fourth, the graph retains only a fixed number of nearest pedestrians and uses a follower-centered star topology. This supports efficient inference but may discard relevant long-range interactions and does not explicitly model pedestrian–pedestrian relations or group structures.

Future work will investigate real robot evaluation, uncertainty- and latency-aware target estimation, visual re-identification, sim-to-real adaptation, socially adaptive rewards, and scalable graph representations incorporating group behavior and broader crowd interactions.

## 7. Conclusions

This paper proposed PPO-GAT-Follow, an interaction-aware reinforcement learning framework for robot person following in dense crowds. The method combines graph attention-based interaction modeling with task-oriented reward shaping to jointly address target maintenance, collision avoidance, rear position accuracy, visibility preservation, close proximity regulation, and motion stability.

In the fixed-route setting with 12 background pedestrians, PPO-GAT-Follow achieves 98.8% task success, a 1.1% collision rate, and an SPL of 0.987. At the random-route training density, it achieves 83.1% task success and an SPL of 0.815, while generalizing without retraining to 6- and 20-pedestrian settings. It consistently outperforms MPC in task success, collision rate, and SPL, and surpasses SARL in the fixed-route and training-density experiments while remaining competitive under high density. The SFM results further show that target attraction and repulsion alone are insufficient for strict rear-following completion.

Ablation studies confirm the contributions of graph-based interaction modeling and balanced reward design. Reward-weight, tolerance shift, multi-seed, and stress analyses demonstrate reasonable robustness to moderate configuration changes, stochastic initialization, target pose noise, and heterogeneous crowd dynamics, although performance degrades under less idealized high-density conditions.

The Gazebo validation further demonstrates integration with localization, UWB-like target-relative pose input, point cloud pedestrian perception, tracking, and velocity control. However, visual target identification, re-identification, tracking failure, and perception-level occlusion recovery remain outside the current scope. Future work will focus on real robot validation, uncertainty-aware perception, human-response modeling, and sim-to-real transfer.

## Figures and Tables

**Table 1:** Architecture of the PPO-GAT-Follow feature extractor.

Component	Value	Description
Graph nodes	$N_{\max}+2$	1 follower, 1 leader, and $N_{\max}$ background pedestrian slots
Node feature dimension	6	Relative position, velocity, distance, and type indicator
Ego feature dimension	10	Following error, target direction, visibility, velocity, heading, previous action, and density
GAT layers	2	Graph attention encoder
GAT heads	4	Multi-head attention in the intermediate layer
GAT hidden dimension	64	Hidden feature dimension
GAT output dimension	64	Follower node graph embedding
Ego hidden dimension	32	Output dimension of the ego MLP branch
Fused feature dimension	128	Input feature dimension for PPO policy and value networks
Policy/value MLP	[256, 256]	Two-layer MLP for actor and critic branches

**Table 2:** Training parameters of PPO-GAT-Follow.

Parameter	Value
Learning rate	$3\times 10^{-4}$
Rollout steps	512
Batch size	256
Optimization epochs per update	10
Discount factor γ	0.99
GAE parameter λ	0.95
Clip range	0.2
Entropy coefficient	0.01
Policy/value MLP	[256, 256]
Parallel environments	8
Total training steps	2,000,000
Main training seed	42
Evaluation frequency	50,000 steps
Validation episodes during training	10
Checkpoint frequency	50,000 steps

**Table 3:** Agent configuration in the RPF environment.

Agent Type	ID	Radius	Behavior and Parameters
Follower	0	0.35m	Controlled by the tested planner or learned policy; each velocity component capped at 1.2m/s
Leader	1	0.28m	RVO controller; $v_{\max}=1.0\;m/s$; acceleration limit $1.0\;m/s^{2}$; avoidance factor 2.0; goal threshold 0.3m
Background pedestrian	2~$\left(N_{c}+1\right)$	0.25m	RVO loop motion between predefined waypoints, producing crossing and occlusion patterns

**Table 4:** Crowd-density settings used in the simulation experiments.

Setting	Background Pedestrians	Total Agents	Purpose
Low density	6	8	Low-density evaluation
Medium density	12	14	Default benchmark
High density	20	22	Dense-crowd stress testing

**Table 5:** Visibility and task completion parameters.

Parameter	Value
Visibility margin	0.05m
Search time limit	5.0s
Desired rear-following distance	1.0m
Following position tolerance	0.45m
Maximum episode length	700 steps

**Table 6:** Training and testing settings for the fixed-route experiment.

Item	Value
Training steps	2,000,000
Background pedestrians	12
Testing seeds	0, 200, 400, …, 1800
Episodes per seed	200

**Table 7:** Training and testing settings for the random-route experiment.

Item	Value
Leader path length	6–16m
Boundary margin	2m
Follower initialization	Approximately 1.8m behind the leader
Training steps	2,000,000
Testing seeds	0, 200, 400, …, 1800
Episodes per seed	200

**Table 8:** Ablation variants of PPO-GAT-Follow.

Type	Variant	Modification
Full model	PPO-GAT-Follow	Two-layer GAT with the full reward design
Structure	PPO-MLP-Follow	Replace graph observation with flat vector observation and remove GAT
Structure	GAT-1Layer	Use a one-layer GAT encoder instead of the default two-layer encoder
Reward	w/o visibility	Remove the visibility reward term
Reward	w/o social zone	Remove the proximity penalty term
Reward	w/o behind	Remove the rear-following reward term

**Table 9:** Fixed-route performance with 12 background pedestrians (10 seeds × 200 episodes).

Method	Task (%)	Coll. (%)	SPL	Vis.	Search	Path (m)	Time (s)	Close Proximity Time (s)
MPC	87.9±1.8	11.9±1.8	0.783±0.021	0.888±0.007	0.947±0.018	21.3±0.3	20.8±0.3	6.88±0.22
DWA	35.7±2.4	64.2±2.2	0.323±0.024	0.848±0.004	0.522±0.020	14.4±0.2	15.8±0.3	5.98±0.23
SFM	0.0±0.0	8.1±2.0	0.000±0.000	0.997±0.001	0.987±0.009	77.6±1.4	65.2±1.2	54.42±1.13
SARL	94.3±1.8	5.4±1.9	0.941±0.018	0.997±0.001	0.987±0.006	19.9±0.3	16.7±0.3	5.32±0.12
PPO-GAT-Follow (Ours)	98.8±0.9	1.1±0.9	0.987±0.008	0.999±0.001	0.998±0.002	19.8±0.0	16.1±0.1	15.48±0.09

*Note:* Bold values indicate the best numerical value in each metric column. Path length, navigation time, and close-proximity time should be interpreted jointly with the task success rate, since failed episodes may terminate prematurely.

**Table 10:** Random-route performance under different numbers of background pedestrians (10 seeds × 200 episodes, random leader layout). PPO-GAT-Follow is trained with 12 background pedestrians and tested at all densities without retraining.

Density	Method	Task (%)	Coll. (%)	SPL	Vis.	Search	Path (m)	Time (s)	Close Proximity Time (s)
Low (6 background pedestrians)	MPC	77.6±2.2	22.1±2.3	0.712±0.021	0.960±0.013	0.985±0.012	14.2±0.5	14.2±0.5	5.03±0.48
	DWA	67.7±3.0	31.9±3.0	0.667±0.031	0.892±0.013	0.911±0.019	10.7±0.3	17.7±0.6	4.25±0.30
	SFM	0.0±0.0	50.8±3.5	0.000±0.000	0.975±0.013	0.954±0.015	57.0±1.9	48.4±1.6	42.88±1.49
	SARL	88.1±1.4	10.5±1.1	0.772±0.018	0.958±0.013	0.963±0.013	14.5±0.3	15.3±0.5	3.85±0.30
	PPO-GAT-Follow (Ours)	92.0±1.7	7.8±1.7	0.912±0.016	0.978±0.012	0.976±0.011	11.2±0.2	10.1±0.2	7.89±0.22
Medium (12 background pedestrians)	MPC	64.8±3.5	34.9±3.3	0.548±0.030	0.885±0.014	0.953±0.020	15.5±0.6	16.0±0.6	6.70±0.58
	DWA	51.2±4.7	48.6±4.8	0.495±0.048	0.805±0.013	0.836±0.015	10.0±0.2	15.9±0.4	4.97±0.29
	SFM	0.0±0.0	68.0±2.8	0.000±0.000	0.939±0.016	0.918±0.014	43.9±1.6	37.6±1.4	32.82±1.37
	SARL	77.6±3.3	21.7±3.4	0.690±0.029	0.915±0.015	0.930±0.016	13.4±0.5	13.6±0.6	5.21±0.39
	PPO-GAT-Follow (Ours)	83.1±1.9	16.7±1.9	0.815±0.017	0.952±0.014	0.954±0.014	10.8±0.3	10.1±0.4	7.87±0.22
High (20 background pedestrians)	MPC	47.2±3.0	52.6±2.9	0.369±0.017	0.818±0.020	0.936±0.015	16.9±0.7	18.2±0.8	8.49±0.49
	DWA	36.4±4.7	63.5±4.7	0.347±0.046	0.734±0.018	0.758±0.026	9.0±0.4	13.7±0.6	5.57±0.23
	SFM	0.0±0.0	85.3±1.6	0.000±0.000	0.899±0.022	0.883±0.023	31.1±1.3	27.1±1.1	23.27±1.05
	SARL	66.5±3.4	33.5±3.4	0.601±0.030	0.870±0.025	0.887±0.025	11.9±0.4	11.1±0.4	5.98±0.22
	PPO-GAT-Follow (Ours)	64.8±4.4	33.7±3.9	0.628±0.044	0.904±0.024	0.911±0.019	10.5±0.3	11.5±0.7	7.75±0.38

*Note:* Bold values indicate the best numerical value in each metric column. Path length, navigation time, and close-proximity time should be interpreted jointly with the task success rate, since failed episodes may terminate prematurely.

**Table 11:** Structural ablation under the random-route setting with 12 background pedestrians (10 evaluation seeds × 200 episodes).

Type	Variant	Task (%)	Coll. (%)	SPL	Vis.	Search	Path (m)	Time (s)	Close Proximity Time (s)
Structure	A1: w/o GAT (MLP)	74.0±3.6	25.8±3.6	0.665±0.038	0.954±0.019	0.956±0.019	13.6±1.0	12.8±1.1	12.16±1.12
	A2: 1-layer GAT	78.8±1.9	20.2±1.5	0.769±0.020	0.952±0.017	0.955±0.015	10.9±0.3	10.0±0.6	8.42±0.30
	A0: Full (Ours)	83.1±1.9	16.7±1.9	0.815±0.017	0.952±0.014	0.954±0.014	10.8±0.3	10.1±0.4	7.87±0.22

*Note:* Bold values indicate the best numerical result among the structural variants.

**Table 12:** Reward ablation under the random-route setting with 12 background pedestrians (10 evaluation seeds × 200 episodes).

Type	Variant	Task (%)	Coll. (%)	SPL	Vis.	Search	Path (m)	Time (s)	Close Proximity Time (s)
Reward	R1: w/o visibility	80.5±3.6	18.8±3.6	0.788±0.036	0.942±0.018	0.950±0.019	10.8±0.3	10.7±0.5	7.96±0.31
	R2: w/o social zone	78.0±2.8	20.5±2.6	0.762±0.027	0.949±0.020	0.946±0.021	10.9±0.2	12.3±0.6	8.44±0.28
	R3: w/o behind reward	79.2±2.3	19.6±2.4	0.775±0.022	0.947±0.018	0.949±0.022	11.0±0.3	11.6±0.5	8.17±0.28
	R0: Full (Ours)	83.1±1.9	16.7±1.9	0.815±0.017	0.952±0.014	0.954±0.014	10.8±0.3	10.1±0.4	7.87±0.22

*Note:* Bold values indicate the best numerical result among the reward variants.

**Table 13:** Reward-weight sensitivity of PPO-GAT-Follow under the random-route setting with 12 background pedestrians (10 evaluation seeds × 200 episodes). Each variant is independently trained for 2 million steps, while Base reuses the main checkpoint. The directed variants increase the corresponding dense reward group by 20%. Random-perturb multiplies all dense reward weights by factors sampled from Uniform(0.8,1.2) and fixed for each training run.

Setting	Task (%)	Coll. (%)	SPL	Vis.	Search	Path (m)	Time (s)	Close Proximity Time (s)
Base	83.1±1.9	16.7±1.9	0.815±0.017	0.952±0.014	0.954±0.014	10.8±0.3	10.1±0.4	7.87±0.22
Target-heavy (+20%)	73.2±2.3	23.7±2.6	0.712±0.025	0.943±0.019	0.945±0.018	10.7±0.3	12.2±0.6	7.78±0.27
Safety-heavy (+20%)	65.0±3.6	25.4±3.2	0.634±0.036	0.926±0.017	0.950±0.016	10.8±0.3	18.4±0.9	8.72±0.30
Visibility-heavy (+20%)	81.0±2.6	16.5±2.5	0.798±0.023	0.955±0.017	0.960±0.017	10.7±0.3	10.9±0.5	7.74±0.29
Random-perturb	80.2±3.1	18.1±3.0	0.790±0.030	0.951±0.020	0.957±0.019	10.7±0.3	10.8±0.5	8.17±0.25

*Note:* Bold values indicate the best numerical result among the evaluated reward-weight settings.

**Table 14:** Tolerance-shift robustness of PPO-GAT-Follow under the random-route setting with 12 background pedestrians (10 evaluation seeds × 200 episodes). The trained checkpoint is fixed, and only the final following position tolerance is varied. The original training and main-table tolerance is 0.45m.

Tolerance	Task (%)	Coll. (%)	SPL	Vis.	Search	Path (m)	Time (s)	Close Proximity Time (s)
0.35m (strict)	63.1±2.7	32.2±2.7	0.610±0.028	0.953±0.015	0.947±0.013	11.6±0.5	18.1±1.2	15.84±0.95
**0.45m (original)**	83.1±1.9	16.7±1.9	0.815±0.017	0.952±0.014	0.954±0.014	10.8±0.3	10.1±0.4	7.87±0.22
0.50m (relaxed)	83.6±1.6	16.2±1.6	0.820±0.015	0.952±0.015	0.954±0.014	10.8±0.3	10.0±0.4	7.78±0.24
0.60m (relaxed)	84.0±1.8	15.8±1.7	0.825±0.015	0.952±0.015	0.954±0.014	10.8±0.3	10.0±0.4	7.71±0.25

*Note:* The 0.45m setting is the original training and evaluation tolerance and is highlighted only for reference. The other rows represent criterion-shift evaluations under stricter or more relaxed tolerances and are not ranked as better or worse configurations.

**Table 15:** Training seed reliability of PPO-GAT-Follow under the random-route setting with 12 background pedestrians. Each policy is independently trained for 2 million steps and evaluated with 10 evaluation seeds × 200 episodes. Seed 42 corresponds to the main checkpoint. The final row reports the mean ± standard deviation across five independent training seeds.

Train Seed	Task (%)	Coll. (%)	SPL	Vis.	Search	Path (m)	Time (s)	Close Proximity Time (s)
**42 (Main checkpoint)**	83.1±1.9	16.7±1.9	0.815±0.017	0.952±0.014	0.954±0.014	10.8±0.3	10.1±0.4	7.87±0.22
142	75.5±3.8	20.4±2.9	0.720±0.040	0.948±0.018	0.939±0.028	12.7±0.5	13.3±0.9	7.79±0.34
242	80.0±1.9	18.9±1.9	0.785±0.019	0.952±0.019	0.959±0.017	11.1±0.4	10.9±0.6	7.77±0.29
342	77.2±2.7	21.6±3.0	0.751±0.025	0.953±0.019	0.961±0.019	10.7±0.5	10.2±0.7	8.22±0.48
442	82.5±3.5	17.4±3.5	0.811±0.035	0.951±0.019	0.950±0.023	10.8±0.3	9.6±0.3	7.32±0.23
Mean ± std (5 seeds)	79.7±3.3	19.0±2.1	0.777±0.041	0.951±0.002	0.952±0.009	11.2±0.8	10.8±1.5	7.80±0.32

*Note:* Seed 42 corresponds to the main checkpoint used in the primary experiments and is highlighted in bold only for identification. Bold formatting is not used to rank the individual training seeds because this experiment evaluates training reproducibility rather than model selection.

**Table 16:** Stress robustness of PPO-GAT-Follow under the original and stress settings.

Density	Setting	Task (%)	Coll. (%)	SPL	Vis.	Search	Path (m)	Time (s)	Close Proximity Time (s)
12 background pedestrians	Original random route	83.1±1.9	16.7±1.9	0.815±0.017	0.952±0.014	0.954±0.014	10.8±0.3	10.1±0.4	7.87±0.22
	Stress setting	75.0±2.6	24.4±2.6	0.735±0.026	0.948±0.014	0.940±0.016	10.8±0.3	10.1±0.4	7.67±0.29
20 background pedestrians	Original random route	64.8±4.4	33.7±3.9	0.628±0.044	0.904±0.024	0.911±0.019	10.5±0.3	11.5±0.7	7.75±0.38
	Stress setting	52.1±4.0	46.5±3.5	0.509±0.039	0.889±0.016	0.887±0.015	9.7±0.2	10.9±0.4	6.93±0.32

## Data Availability

The data presented in this study are available from the corresponding author upon reasonable request.

## Supplementary Materials

The following supporting information can be downloaded at: https://www.mdpi.com/article/10.3390/s26154711/s1, Video S1: Dynamic demonstrations of PPO-GAT-Follow in the IR-SIM and Gazebo environments.
